# Inhibition of Fatty Acid Metabolism Reduces Human Myeloma Cells Proliferation

**DOI:** 10.1371/journal.pone.0046484

**Published:** 2012-09-28

**Authors:** José Manuel Tirado-Vélez, Insaf Joumady, Ana Sáez-Benito, Irene Cózar-Castellano, Germán Perdomo

**Affiliations:** 1 Unidad de Investigación, Hospital Universitario Puerta del Mar, Cádiz, Spain; 2 Clinical Laboratory Department, Hospital Universitario Puerta del Mar, Cádiz, Spain; Visva Bharati University, India

## Abstract

Multiple myeloma is a haematological malignancy characterized by the clonal proliferation of plasma cells. It has been proposed that targeting cancer cell metabolism would provide a new selective anticancer therapeutic strategy. In this work, we tested the hypothesis that inhibition of β-oxidation and de novo fatty acid synthesis would reduce cell proliferation in human myeloma cells. We evaluated the effect of etomoxir and orlistat on fatty acid metabolism, glucose metabolism, cell cycle distribution, proliferation, cell death and expression of G1/S phase regulatory proteins in myeloma cells. Etomoxir and orlistat inhibited β-oxidation and de novo fatty acid synthesis respectively in myeloma cells, without altering significantly glucose metabolism. These effects were associated with reduced cell viability and cell cycle arrest in G0/G1. Specifically, etomoxir and orlistat reduced by 40–70% myeloma cells proliferation. The combination of etomoxir and orlistat resulted in an additive inhibitory effect on cell proliferation. Orlistat induced apoptosis and sensitized RPMI-8226 cells to apoptosis induction by bortezomib, whereas apoptosis was not altered by etomoxir. Finally, the inhibitory effect of both drugs on cell proliferation was associated with reduced p21 protein levels and phosphorylation levels of retinoblastoma protein. In conclusion, inhibition of fatty acid metabolism represents a potential therapeutic approach to treat human multiple myeloma.

## Introduction

Oncogenic transformation of normal cells into tumor cells involves a well-orchestrated metabolic reprogramming of glucose and fatty acid metabolism. In addition, tumor cells exhibit a coordinated regulation between metabolic pathways and cell growth signaling pathways in order to sustain an elevated rate of cell proliferation and survival, suggesting that activation of oncogenes and/or inactivation of tumor suppressors participate in the metabolic reprogramming of tumor cells [1]. Therefore, understanding tumor cell metabolism would provide a rationale for the design of new therapeutic targets in the area of cancer research.

Tumor cells satisfy the bioenergetic and biosynthetic demands for cell growth and survival using glucose [1]. Glucose is consumed at a high rate to produce lactate and ATP even in the presence of oxygen. This metabolic adaptation from oxidative to glycolytic metabolism, known as the “Warburg effect”, was first reported by Otto Warburg in the 1920s [2], [3]. At first glance, these metabolic alterations in glucose metabolism seems to be inappropriate to sustain tumor cells growth and survival, because aerobic glycolysis is 18-fold less efficient to produce ATP than oxidative phosphorylation. However, tumor cells overcome this limitation increasing the glycolytic flux of glucose by a mechanism that at least involves upregulation of glucose transporter 1 (GLUT1) [4].

Another hallmark of tumor cells is an elevated rate of lipogenesis, which is mainly supported by de novo fatty acid synthesis (FAS) rather than by dietary fatty acids [5]. In fact, neoplastic cells exhibit an elevated expression and activity of fatty acid synthase (FASN; a multi-enzyme that catalyzes the conversion of malonyl-CoA into fatty acids through de novo fatty acid synthesis pathway) [5]. The newly synthesized free fatty acids can be esterified and stored into neutral lipids (such as monoacylglycerol, diacylglycerol and triacylglycerol), transformed into membrane lipids (such as cholesterol or phospholipids) and converted into signaling lipids (such as phosphatidic acid, lysophosphatidic acid or prostaglandin E2) [6]. In addition, endogenous fatty acid catabolism through β-oxidation can provide an alternative pathway to support cancer cell growth and survival. To this end, fatty acids are transported from the cytosol to the mitochondrial matrix by the carnitine palmitoyl transferase (CPT) system. The first and most tightly regulated component of the CPT system (CPT I; carnitine palmitoyl transferase I) is potently inhibited by malonyl-CoA, a key intermediary in the opposing pathway of de novo fatty acid synthesis described above [7]. Interestingly, mitochondrial long-chain fatty acid oxidation (FAO) confers an alternative route for energy provision [8] and chemoresistance to cancer cells [9].

The mechanisms that regulate cell cycle entry, progression and clonal expansion in multiple myeloma cells are incompletely understood. In tumor cells, coordinated regulation between metabolic alterations in glucose and fatty acid metabolism, and intracellular energy and nutrient sensors, trigger signalling pathways involved in cell growth and survival. These pathways lead to an activation of D-type cyclins (such as cyclin D1 and cyclin D2), a class of proteins without catalytic activity. D-type cyclins bind and activate cyclin dependent kinases 4 and 6 (CDK4 and CDK6) enabling an active complex cyclin-CDK that phosphorylates the retinoblastoma protein (pRb; the gatekeeper of G_1_/S transition). In the absence of a mitogenic stimulus, pRb is bound to E2F, a family of transcriptional activators and repressors that control cell cycle progression during G_1_/S phase. Upon phosphorylation of pRb by the active cyclin-CDK complexes, E2F transcriptional factors are unleashed allowing cell progress toward S phase [10].

To our best knowledge, fatty acid metabolism and the therapeutic benefits of inhibiting these metabolic pathways has not been extensively investigated in multiple myeloma. Therefore, we have first characterized fatty acid metabolism and the impact of its pharmacological inhibition on glucose metabolism in myeloma cells. Second, we investigated the therapeutic benefits of fatty acid metabolism inhibition in myeloma cells using etomoxir (a specific inhibitor of CPT I, which has been used in clinical trials in patients with chronic heart failure) [11], [12] and orlistat (a Food and Drug Administration (FDA)-approved anti-obesity drug that specifically inhibits FASN activity) [13], [14]. Third, we investigated the impact of fatty acid metabolism inhibition on the regulation of G_1_/S phase in human myeloma cells. We demonstrated that etomoxir and orlistat significantly reduced proliferation of human myeloma cells by a mechanism that at least involves reduced protein levels of p21 and phosphorylation of retinoblastoma protein (pRb). Finally, our findings lend support to the clinical evaluation of fatty acid metabolism inhibitors for the treatment of multiple myeloma patients.

## Results

### Sensitivity of Myeloma Cell Lines to Inhibitors of Fatty Acid Metabolism

The sensitivity of myeloma cells to inhibitors of fatty acid metabolism have not been investigated in myeloma cells. To this end, we first characterized the fatty acid oxidation and de novo fatty acid synthesis capacity in three human myeloma cell lines (RPMI-8226, NCI-H929 and U-266B1); and second, we investigated the sensitivity of myeloma cells to fatty acid oxidation and de novo fatty acid synthesis inhibition.

**Figure 1:**
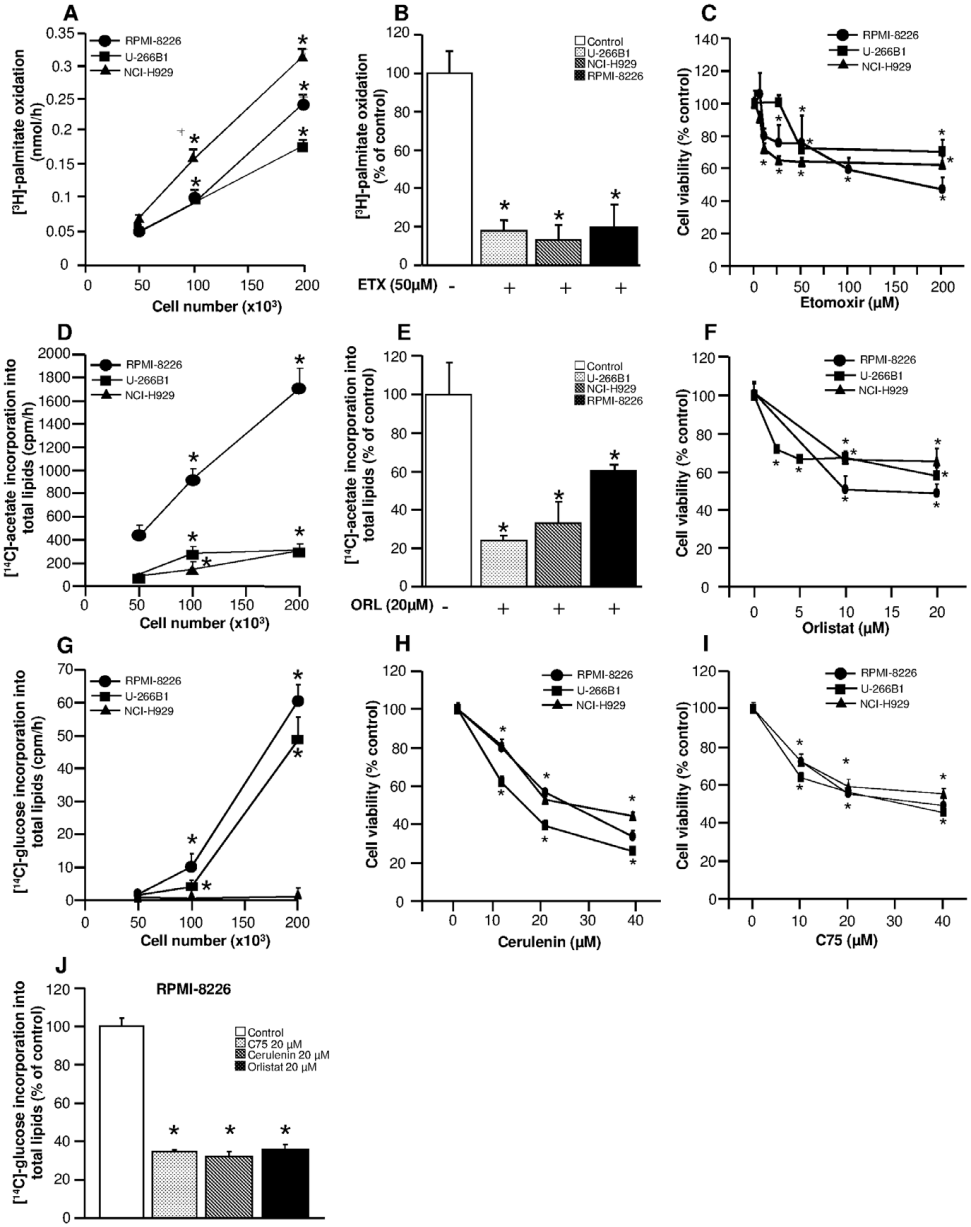
Effects of etomoxir and orlistat on fatty acid metabolism and cell viability in myeloma cell lines. (**A**) Mitochondrial β-oxidation of exogenous fatty acids in myeloma cell lines. RPMI-8226, NCI-H929 and U-266B1 cells were preincubated with tritiated palmitate for 18 h for measurement of fatty acid oxidation rates. Data are represented as means ± SEM for four to six independent experiments for each cell line. *p<0.05 versus 50×10^3^ cells. (**B**) Etomoxir inhibits mitochondrial β-oxidation in myeloma cells. RPMI-8226, NCI-H929 and U-266B1 cells were preincubated in the presence or absence of 50 µM etomoxir for 18 h and β-oxidation was determined as described in “Materials and Methods”. Data are represented as means ± SEM for four to six independent experiments for each cell line. *p<0.05 versus untreated cells. (**C**) Etomoxir reduces cell viability in myeloma cells. Cell viability was assessed after 18 h treatment with the indicated doses of etomoxir. Results are expressed as percentage of values in untreated cells (n = 4–6 independent experiments). *p<0.05 versus untreated cells. (**D**) De novo lipid synthesis from acetate as carbon source in myeloma cell lines. RPMI-8226, NCI-H929 and U-266B1 cells were preincubated with [^14^C]-acetate for 24 h for measurement of [^14^C]-acetate incorporation into total lipids. Data are represented as means ± SEM for four to six independent experiments for each cell line. *p<0.05 versus 50×10^3^ cells. (**E**) Orlistat inhibits de novo lipogenesis in myeloma cells. RPMI-8226, NCI-H929 and U-266B1 cells were preincubated in the presence or absence of 20 µM orlistat for 24 h, and [^14^C]-acetate incorporation into total lipids was determined as described in “Materials and Methods” section. Data are represented as means ± SEM for four to six independent experiments for each cell line. *p<0.05 versus untreated cells. (**F**) Orlistat reduces cell viability in myeloma cells. Cell viability was assessed after 24 h treatment with indicated doses of orlistat. Results are expressed as percentage of values in untreated cells (n = 4–6 independent experiments). *p<0.05 versus untreated cells. (**G**) De novo lipid synthesis from glucose as carbon source in myeloma cell lines. RPMI-8226, NCI-H929 and U-266B1 cells were preincubated with [^14^C]-glucose for 24 h for measurement of [^14^C]-glucose incorporation into total lipids. Data are represented as means ± SEM for three independent experiments for each cell line. *p<0.05 versus 50×10^3^ cells. (**H**) Cerulenin reduces cell viability in myeloma cells. Cell viability was assessed after 24 h treatment with indicated doses of cerulenin. Results are expressed as percentage of values in untreated cells (n = 9 independent experiments). *p<0.05 versus untreated cells. (**I**) C75 reduces cell viability in myeloma cells. Cell viability was assessed after 24 h treatment with indicated doses of C75. Results are expressed as percentage of values in untreated cells (n = 9 independent experiments). *p<0.05 versus untreated cells. (**J**) Cerulenin, C75 or orlistat inhibit de novo lipogenesis in myeloma cells. RPMI-8226 cells were preincubated in the presence or absence of 20 µM cerulenin, 20 µM C75 or 20 µM orlistat for 24 h and [^14^C]-glucose incorporation into total lipids was determined as described in “Materials and Methods” section. Data are represented as means ± SEM for nine independent experiments for each cell line. *p<0.05 versus untreated cells.

To determine the fatty acid oxidation capacity, human myeloma cells were incubated with [^3^H]-palmitate for 18 h. As shown in Figure 1A, myeloma cells exhibited a linear relationship between cell number and β-oxidation, demonstrating that myeloma cells can oxidize exogenous fatty acids. Then, we used etomoxir, an irreversible inhibitor of CPT I, as an inhibitor of β-oxidation. Incubation of myeloma cells with etomoxir at 50 µM for 18 h reduced by 80–90% β-oxidation capacity in myeloma cells (Figure 1B). Finally, we investigated the impact of β-oxidation inhibition on viability of myeloma cells. Myeloma cells were incubated with a range of etomoxir concentrations for 18 h and cell viability was assayed. As shown in Figure 1C, etomoxir reduced by 20–40% cell viability at doses between 5–25 µM, reaching a plateau at doses between 25–200 µM, indicating that myeloma cells rely on β-oxidation capacity to sustain cell viability.

To determine de novo fatty acid lipogenesis capacity, human myeloma cells were incubated with [^14^C]-acetate for 24 h. Myeloma cells showed a linear relationship between cell number and lipogenesis, demonstrating a metabolic capacity to synthesize lipids in these cells (Figure 1D). The lipogenic capacity of U-266B1 and NCI-H929 cells was 3–6-fold lower than in RPMI-8226 cells using [^14^C]-acetate as carbon source (Figure 1D). Orlistat is an anti-obesity agent that inhibits lipase/fatty acid synthase activity [15]. Incubation of myeloma cells with orlistat at 20 µM for 24 h reduced by ∼50% de novo lipogenesis (Figure 1E). In addition, orlistat reduced by 20–40% cell viability at doses between 2,5–10 µM, reaching a plateau at doses between 10–20 µM (Figure 1F).

To further investigate the “de novo” fatty acid lipogenesis capacity, human myeloma cells were incubated with [^14^C]-glucose for 24 h. RPMI-8226 and U-266B1 cells showed a linear relationship between cell number and lipogenesis, demonstrating a metabolic capacity to synthesize lipids from glucose in these cells (Figure 1G). The RPMI-8226 cell line showed the highest lipogenic capacity from both acetate and glucose carbon sources (Figures 1D and 1G). Finally, the lipogenic capacity of NCI-H929 cells was 2–60-fold lower than in RPMI-8226 and U-266B1 cells using [^14^C]-glucose as carbon source (Figure 1G).

Cerulenin and its synthetic derivative C75 are known inhibitors of FASN [16]. Incubation of myeloma cells with cerulenin reduced by ∼60–70% cell viability at doses between 10–40 µM (Figure 1H). Likewise, C75 reduced by ∼50% cell viability at doses between 10–40 µM (Figure 1I). Finally, incubation of myeloma cells with cerulenin, C75 or orlistat at 20 µM for 24 h reduced by ∼60% de novo lipogenesis, using glucose as a carbon source (Figure 1J).Taken together, these results indicate that cell viability of human myeloma cells depend on de novo lipogenesis.

### Effect of Etomoxir and Orlistat on Glucose Metabolism in Myeloma Cells

Because fatty acid oxidation and de novo lipogenesis inhibition reduced cell viability in myeloma cells, we sought to investigate the impact of fatty acid oxidation and de novo lipogenesis inhibition on glucose metabolism in myeloma cells.

**Figure 2:**
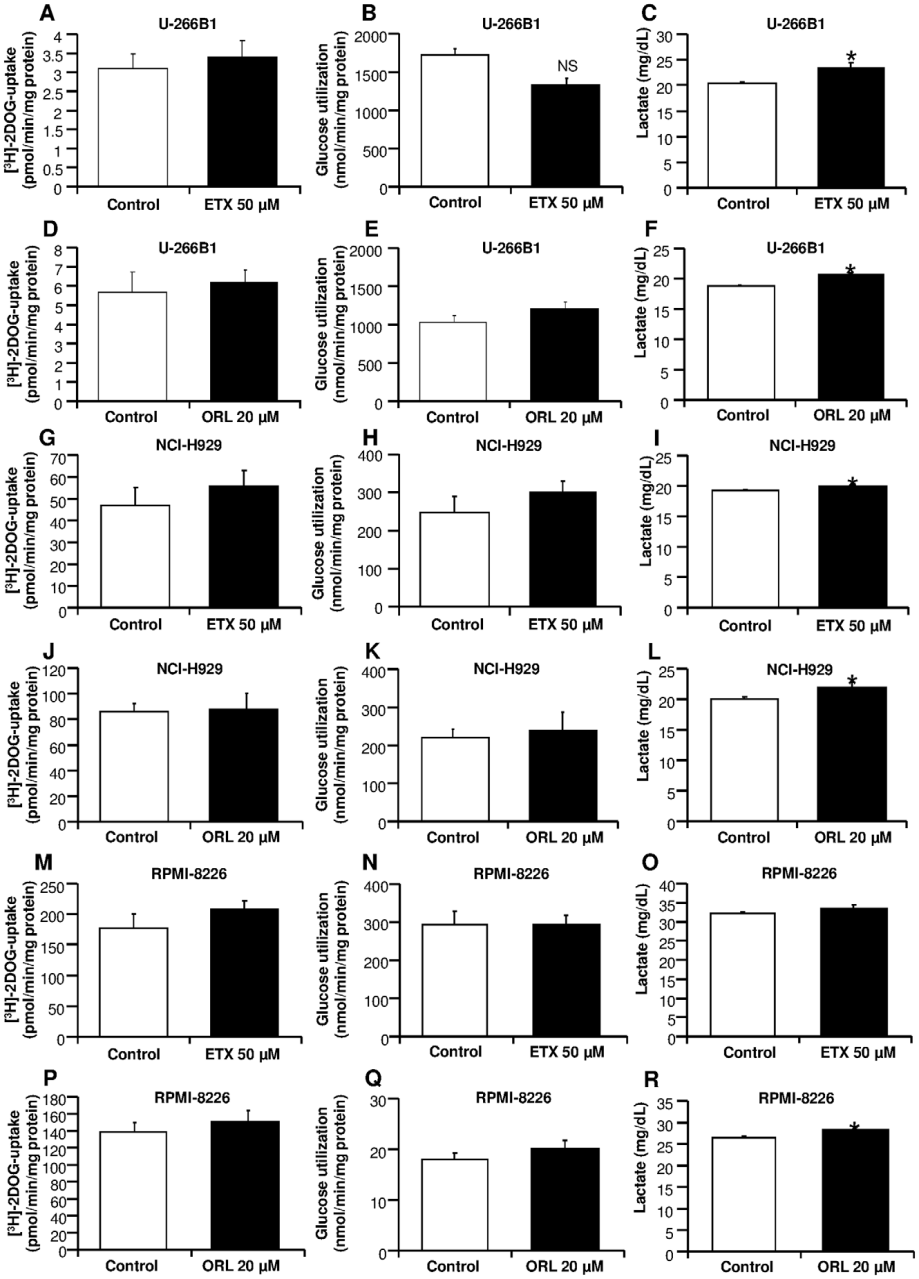
Effects of etomoxir and orlistat on glucose metabolism in myeloma cell lines. Myeloma cells were preincubated in the presence or absence of 50 µM etomoxir or 20 µM orlistat for 18 h and 24 h respectively before measurement of glucose uptake, glycolysis, or lactate production as described in “Materials and Methods” section. Effects of etomoxir (**A–C**) or orlistat (**D–F**) on glucose metabolism in U-266B1 cells. Effects of etomoxir (**G–I**) or orlistat (**J–L**) on glucose metabolism in NCI-H929 cells. Effects of etomoxir (**M–O**) or orlistat (**P–R**) on glucose metabolism in RPMI-8226 cells. Data are represented as means ± SEM for four to six independent experiments in triplicate. *p<0.05 versus untreated cells.

To evaluate glucose uptake, we treated myeloma cells with 50 µM etomoxir or 20 µM orlistat for 18 h and 24 h respectively. We observed that etomoxir or orlistat treatment did not alter the glucose transport capacity in U-266B1 cells (Figure 2A, D), NCI-H929 cells (Figure 2G,J) and RPMI-8226 cells (Figure 2M,P). To further investigate the impact of etomoxir and orlistat on glucose metabolism, we measured the glucose utilization in myeloma cells treated with etomoxir and orlistat as above. Similarly to glucose uptake, etomoxir and orlistat did not alter glucose utilization capacity in U-266B1 cells (Figure 2B, E), NCI-H929 cells (Figure 2H,K) and RPMI-8226 cells (Figure 2N,Q). Finally, etomoxir treatment increased by ∼10% lactate production in U-266B1 cells (Figure 2C) and NCI-H929 cells (Figure 2I), whereas lactate production remained unchanged in RPMI-8226 cells (Figure 2O). Similarly, orlistat treatment increased by ∼10% lactate production in U-266B1 cells (Figure 2F), NCI-H929 cells (Figure 2L) and RPMI-8226 cells (Figure 2R).

### Etomoxir and Orlistat Halt Cells in G0/G1 Phase in Human Myeloma Cells

**Figure 3:**
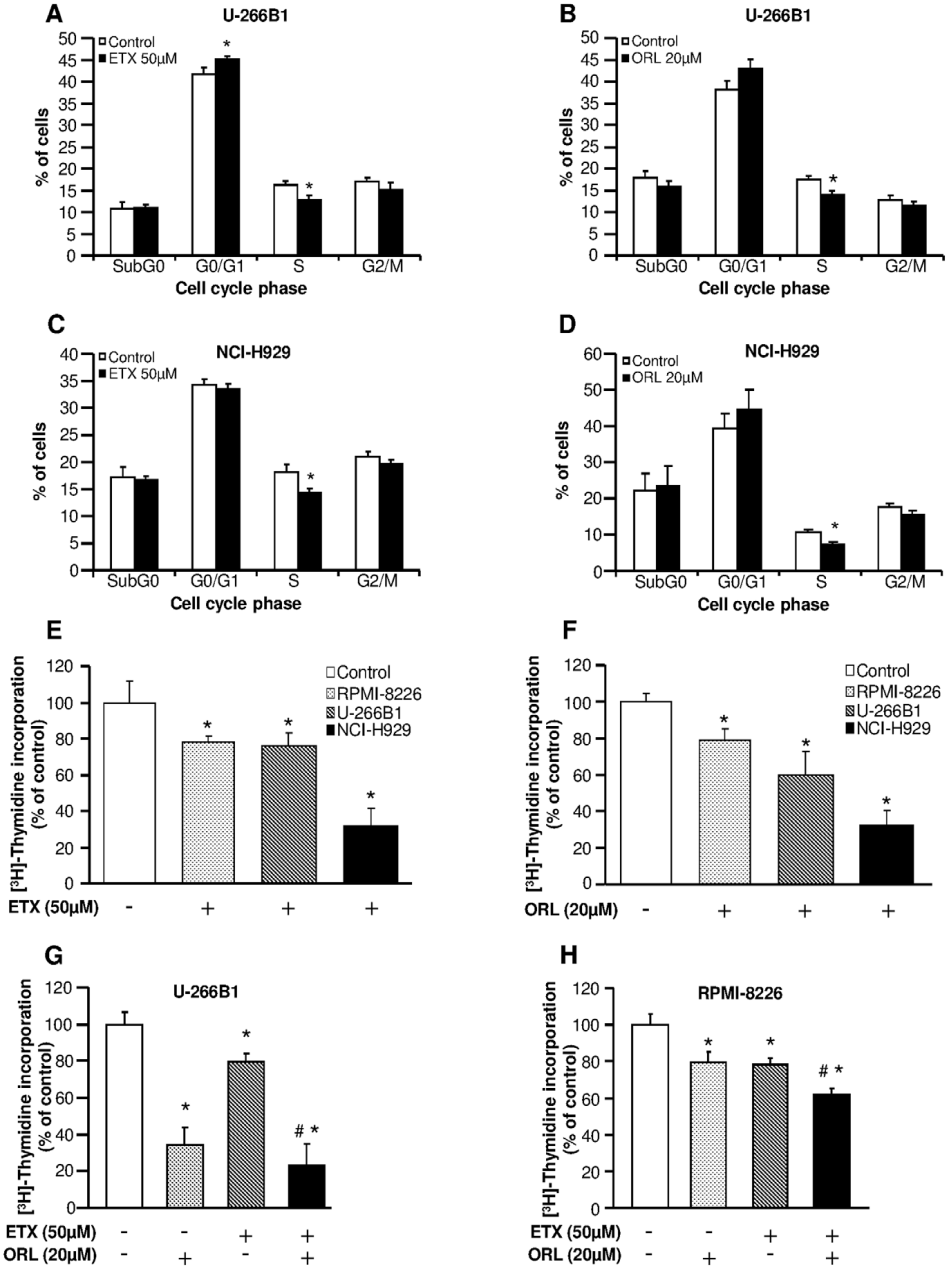
Etomoxir and orlistat inhibit cell cycle progression and decreased proliferation rate in myeloma cells. The effect of etomoxir and orlistat on cell cycle phases was determined in myeloma cells preincubated in the presence or absence of 50 µM etomoxir or 20 µM orlistat for 18 or 24 hours respectively as described in “Materials and Methods” section. (**A,B**) Cell cycle phases in U-266B1 cells. (**C,D**) Cell cycle phases in NCI-H929 cells. Results are means ± SEM for 3 independent experiments in triplicate. *p<0.05 versus untreated cells. The effect of 50 µM etomoxir (**E**), 20 µM orlistat (**F**) on the proliferation rate in RPMI-8226, U-266B1 and NCI-H929 cells was determined by the incorporation of tritiated thymidine assay. Results are means ± SEM for 3–6 independent experiments in triplicate. *p<0.05 versus untreated cells. The effect of 50 µM etomoxir, 20 µM orlistat or the combination of both drugs on the proliferation rate in U-266B1 cells (**G**) and RPMI-8226 cells (**H**) was determined by the incorporation of tritiated thymidine assay. Results are means ± SEM for 3–6 independent experiments in triplicate. *p<0.05 versus untreated cells. ^#^p<0.05 versus etomoxir and orlistat treated cells.

To investigate the mechanism by which etomoxir and orlistat reduce myeloma cells viability, we next examine the effect of etomoxir and orlistat on the cell cycle phases of human myeloma cells. Myeloma cells were preincubated in the presence or absence of 50 µM etomoxir for 18 h, or 20 µM orlistat for 24 h, and cells were harvested for cell cycle analysis using propidium iodide staining. In U-266B1 and NCI-H929 cells, etomoxir and orlistat reduced by ∼5% the fraction of cells in S phase, which corresponds to a relative decreased of ∼20% (Figure 3A–B and 3C–D respectively). In addition, etomoxir increased by ∼10% the fraction of cells in G0/G1 phase in U-266B1 cells, whereas in the presence of orlistat the fraction of cells in G0/G1 remained unchanged (Figure 3A–B). In contrast, etomoxir or orlistat did not alter the fraction of cells in G0/G1 in NCI-H929 cells. Interestingly, the fraction of cells in SubG0 phase remained unchanged in the presence of etomoxir or orlistat (Figure 3A–D) in U-266B1 and NCI-H929 cells. Taken together, these results suggest that the main mechanism by which etomoxir and orlistat reduce cell viability in human myeloma cells is due to inhibition of proliferation, avoiding cells to progress from G0/G1 to S phase.

### Etomoxir and Orlistat Inhibit Proliferation in Myeloma Cells

To test the hypothesis that etomoxir and orlistat inhibit proliferation of myeloma cells, RPMI-8226, U-266B1 and NCI-H929 cells were preincubated in the presence or absence of 50 µM etomoxir or 20 µM orlistat for 18 h and 24 h respectively. Then, [^3^H]-thymidine incorporation into nucleic acids was determined in untreated and treated cells. As shown in Figure 3E–F, etomoxir and orlistat decreased by ∼20–70% proliferation rate in myeloma cells. In addition, etomoxir plus orlistat exhibited an additive effect on proliferation rate in U-266B1 cells (Figure 3G) and RPMI-8226 cells (Figure 3H).

### Orlistat Sensitizes RPMI-8226 Cells to Bortezomib-induced Apoptosis

**Figure 4:**
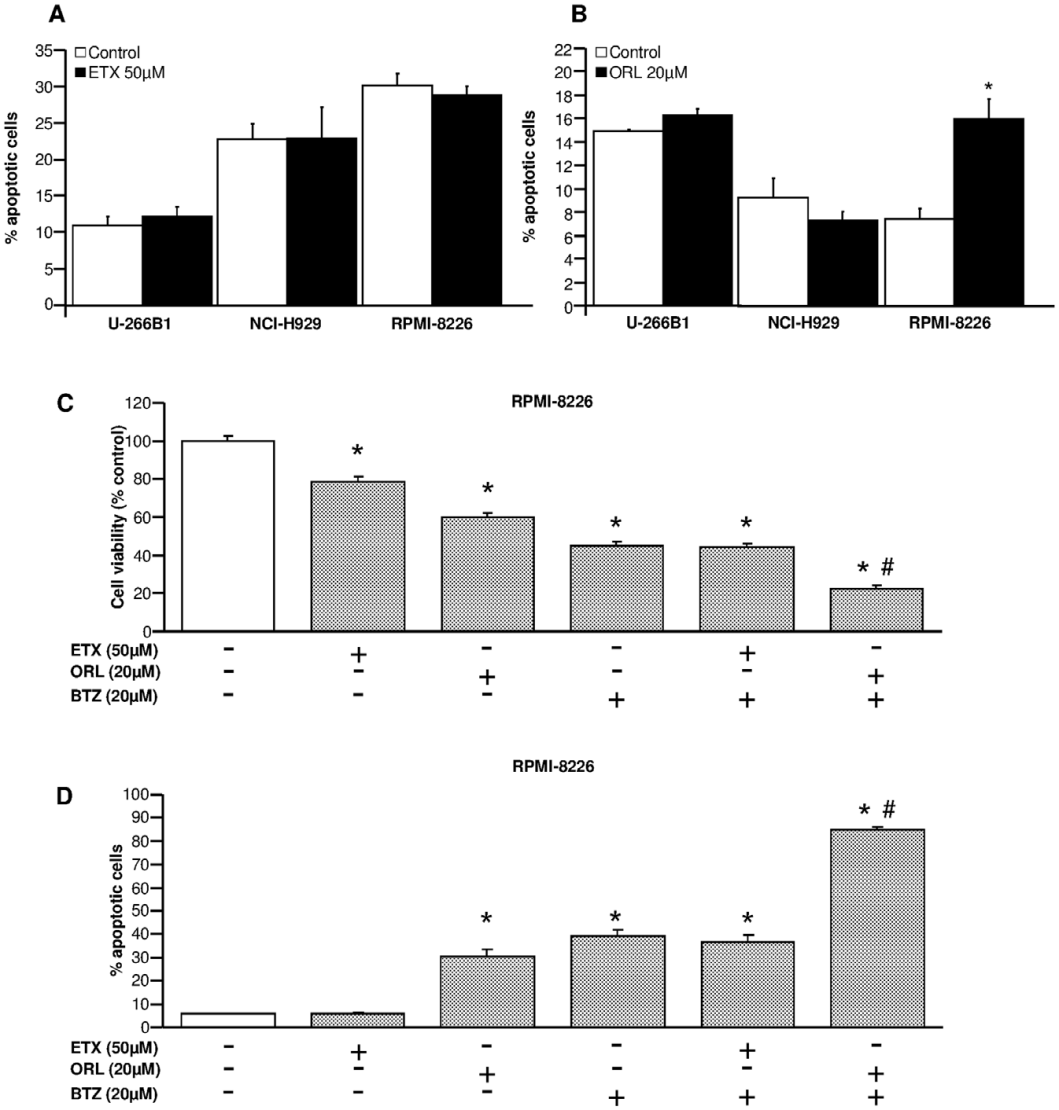
Orlistat enhances the apoptotic effect of bortezomib in RPMI-8266 cells. The effect of etomoxir (**A**) or orlistat (**B**) on apoptosis in U-266B1, NCI-H929 and RPMI-8226 cells was determined by FACS analysis of intracellular cleaved-caspase-3 levels. Cells were preincubated in the presence or absence of 50 µM etomoxir or 20 µM orlistat for 18 h and 24 h respectively, and cleaved-caspase-3 levels were analyzed as described in the “Materials and Methods” section. Results are means ± SEM for 3 independent experiments in triplicate. *p<0.05 versus untreated cells. (**C**) The effect of etomoxir, orlistat or bortezomib on cell viability was analyzed in RPMI-8226 cells. Cells were preincubated in the presence or absence of 50 µM etomoxir, 20 µM orlistat or 20 µM bortezomib for 24 hours. Afterwards, cell viability was determined as described in the “Materials and Methods” section. Results are means ± SEM for 6 independent experiments in triplicate. *p<0.05 versus untreated cells. ^#^p<0.05 versus orlistat or etomoxir alone. (**D**) The effect of etomoxir, orlistat or bortezomib on apoptosis was analyzed in RPMI-8226 cells. Cells were preincubated as described above. Afterwards, the percentage of apoptotic cells was determined by FACS analysis of intracellular cleaved-caspase-3 levels as described above. Results are means ± SEM for 6 independent experiments in triplicate. *p<0.05 versus untreated cells. ^#^p<0.05 versus orlistat or etomoxir alone.

Next, we investigated the effects of inhibition of fatty acid metabolism on apoptosis in U-266B1, NCI-H929 and RPMI-8226 myeloma cells. In figure 4A, we show that 50 µM etomoxir did not alter the apoptotic rate in myeloma cells. However, 20 µM orlistat increased by ∼50% de percentage of apoptotic cells in RPMI-82266 cells (Figure 4B), whereas apoptosis remained unchanged in U-266B1 and NCI-H929 cells (Figure 4B). These data indicate that orlistat induced apoptosis in the most lipogenic cell line.

To further investigate the effects of fatty acid inhibition on apoptosis in myeloma cells, we sought to decipher whether orlistat and etomoxir sensitize RPMI-8226 cells to apoptosis induction by bortezomib. As expected, 50 µM etomoxir, 20 µM orlistat or 20 µM bortezomib decreased cell viability in RPMI-8226 cells by 20%, 40% and 50% respectively (Figure 4C). Interestingly, 20 µM orlistat plus 20 µM bortezomib exhibited an additive effect on reduction of cell viability (∼80%) compared to orlistat or bortezomib alone, which was not present in the combination 50 µM etomoxir plus 20 µM orlistat compared to etomoxir or orlistat alone (Figure 4C). These effects of fatty acid metabolism inhibition on cell viability were paralleled with increased apoptosis in RPMI-8226 cells treated with orlistat and bortezomib. As showed before in figure 4A, 50 µM etomoxir alone did not increase apoptosis in RPMI-8266 cells (Figure 4D). Likewise, 50 µM etomoxir combined with 20 µM bortezomib did not increased the percentage of apoptotic cells compared to bortezomib alone (Figure 4D). Interestingly, cells treated with 20 µM orlistat or 20 µM bortezomib showed an apoptotic rate between 30–40%, whereas the combination of both drugs exhibited an additive effect on apoptosis, reaching ∼85% cell death in RPMI-8226 (Figure 4D). These results indicate that orlistat sensitizes the apoptotic effect of bortezomib on RPMI-8226 cells.

### Etomoxir and Orlistat Decreased p21 and p-pRb Levels in Human Myeloma Cells

**Figure 5:**
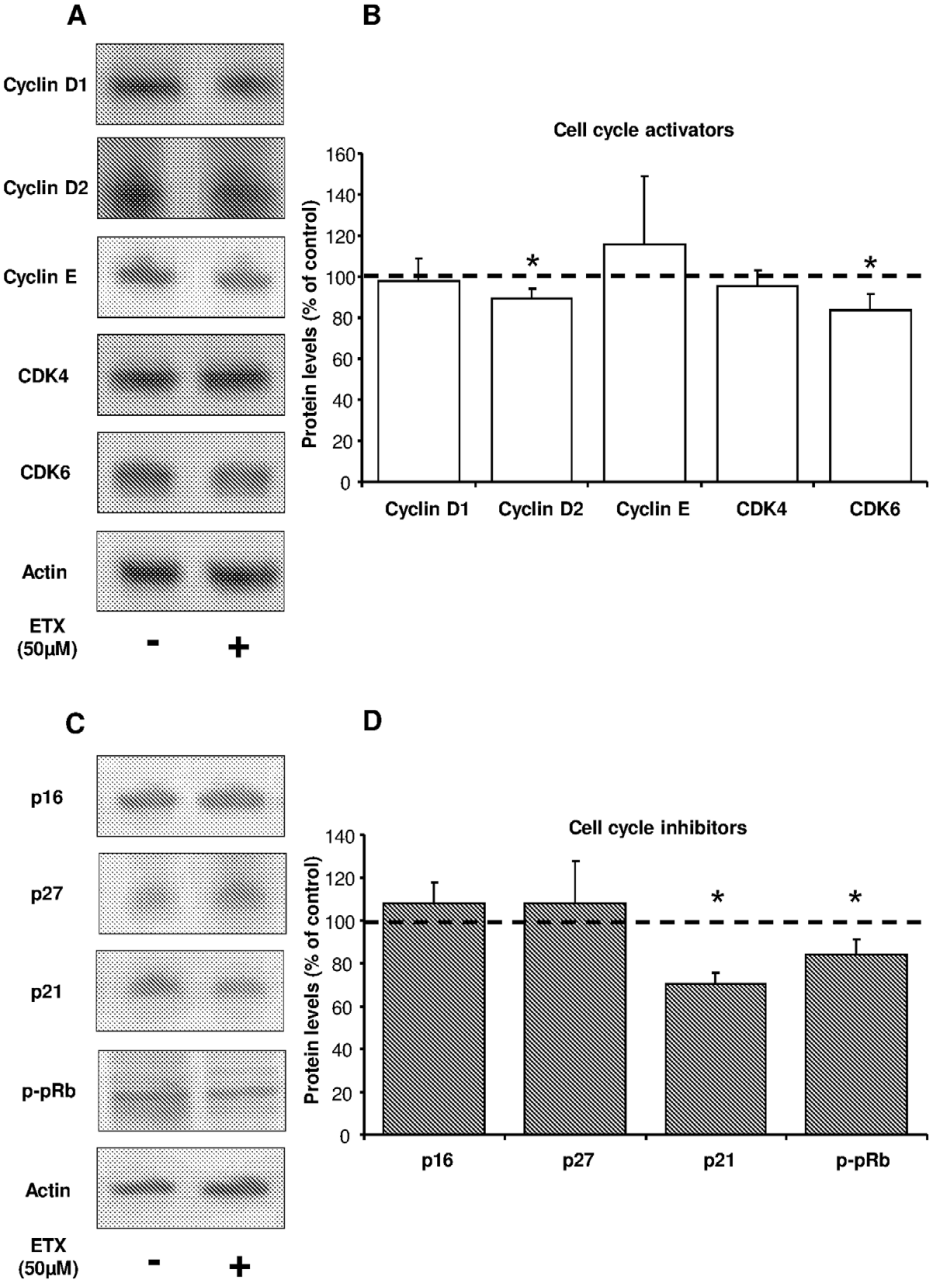
Effects of etomoxir on cell cycle regulatory proteins in myeloma cells. Western blot analysis of cell cycle regulatory proteins from etomoxir-treated myeloma cells. Cells were treated in the presence or absence of 50 µM etomoxir for 18 hours before harvesting the cells, preparation of cell extracts and detection of proteins by specific antibodies. Representative immunoblot of cell cycle activators (**A**) and inhibitors (**C**). Quantification of the total data set expressed as percentage of untreated cells (**B, D**). Mean ± SEM for 4–6 independent experiments. *p<0.05 versus untreated cells.

**Figure 6:**
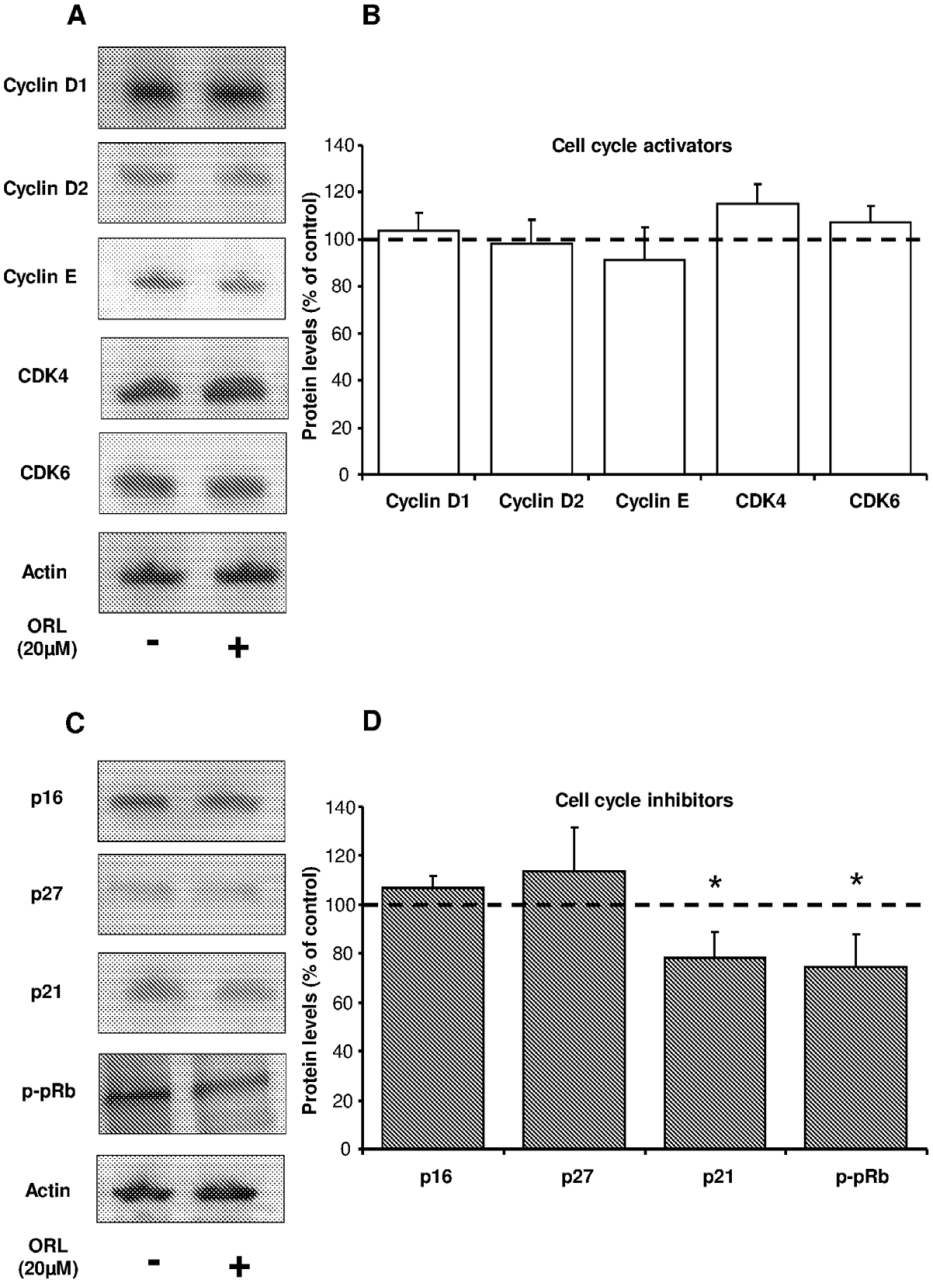
Effects of orlistat on cell cycle regulatory proteins in myeloma cells. Western blot analysis of cell cycle regulatory proteins from orlistat-treated myeloma cells. Cells were treated in the presence or absence of 20 µM orlistat for 24 hours before harvesting the cells, preparation of cell extracts and detection of proteins by specific antibodies. Representative immunoblot of cell cycle activators (**A**) and inhibitors (**C**). Quantification of the total data set expressed as percentage of control (**B, D**). Mean ± SEM for 4–6 independent experiments. *p<0.05 versus untreated cells.

To further investigate the molecular mechanism by which etomoxir and orlistat decreased proliferation in myeloma cells, we analyzed cell cycle proteins involved in the activation or inhibition of cell cycle. As shown in figure 5, myeloma cells treated with 50 µM etomoxir exhibited a significant but modest reduction in the protein levels of cyclin D2 and CDK6. However, p21 and phospho-pRb levels were significantly reduced in comparison to untreated cells. In contrast, cyclin D1, Cyclin E, CDK4, p16 and p27 remained unchanged in response to etomoxir treatment. Likewise, myeloma cells treated with 20 µM orlistat exhibited a significant reduction in p21 and phospho-pRb levels in myeloma cells, whereas cyclin D1, cyclin D2, cyclin E, CDK4, CDK6, p16 and p27 remained unchanged (Figure 6).

## Discussion

In this study, we have tested the hypothesis that inhibition of fatty acid oxidation and de novo fatty acid synthesis would reduce proliferation in human myeloma cells. To this end, we have used two drugs that have undergone clinical trials as treatment for cardiovascular disease and obesity. We demonstrated that etomoxir and orlistat significantly reduced cell viability and proliferation in myeloma cells. Interestingly, orlistat induced apoptosis and sensitized RPMI-8226 cells to bortezomib-induced apoptosis. The molecular mechanisms by which etomoxir and orlistat inhibited proliferation in myeloma cells is associated to reduce p21 protein levels and phosphorylation levels of pRb, two key proteins involved in G_1_/S cell cycle progression.

Etomoxir, a specific and irreversible inhibitor of CPT I, is a clinically well-tolerated drug that has been used in clinical trials in patients with chronic heart failure and diabetes [11], [12], [17], [18]. A few studies reported that etomoxir-mediated inhibition of FAO is associated with inhibition of proliferation and sensitization to apoptosis induction by genotoxic and non-genotoxic drugs in tumor cells [19], [20], [21]. In our study, the main effect of etomoxir on myeloma cells was due to inhibition of proliferation, whereas apoptosis remained unchanged. In addition, we demonstrated that etomoxir and orlistat exhibited an additive effect on inhibition of myeloma cell proliferation.

Likewise, orlistat, a specific inhibitor of FASN currently used in the treatment of obesity, reduced cell proliferation in myeloma cells [14]. Recently, Wang et al. have described that human bone marrow samples from multiple myeloma patients expressed high levels of FASN, whereas peripheral blood mononuclear cells (PBMCs) from healthy donors did not express detectable levels of FASN [22]. It has been described that orlistat is a potent and selective inhibitor of FASN in prostate carcinoma cells and in breast cancer cells [13], [14].

Orlistat is an inhibitor of the thioesterase activity of FASN. Although initially the inhibitory properties of orlistat were demonstrated for pancreatic lipase, a large survey of a number of tumor and normal cell lines has lead to the conclusion that orlistat has no other targets than FASN in tumor cells, and that has minimal effects on the normal cells. Taken together these considerations, it has been proposed orlistat as a compound with therapeutic index sufficient for antitumor therapy [13]. We demonstrated that 10 µM orlistat reduced by ∼30–50% cell viability in myeloma cells. On the other hand, 10 µM cerulenin or 10 µM C75 reduced by ∼20–40% cell viability in myeloma cells. These data indicate that the effect of orlistat on cell viability is similar to cerulenin and C75 FASN inhibitors. Interestingly, orlistat has been proposed as an alternative to cerulenin and its synthetic derivative C75, which inhibits the ketoacyl synthase domain of FASN but also stimulates the carnitine palmitoyltransferase I activity [23].

In addition, we showed that orlistat induces an anti-proliferative effect in myeloma cells. Of note, our observations also demonstrated that this anti-proliferative effect of the inhibition of de novo fatty acid synthesis in the most lipogenic cell line of the three myeloma cell lines used (i.e. RPMI-8266) associated with apoptosis induction. Furthermore, orlistat sensitized RPMI-8226 cells to bortezomib-induced apoptosis. Although the mechanisms underlying these effects of orlistat in myeloma cells remain to be deciphered, and confirmation of these results in freshly isolated human myeloma cells and/or murine myeloma models are warranted, they provide a proof of principle that inhibition of de novo fatty acid metabolism can be a pharmacological target for agents that activate the apoptotic pathway in myeloma cells and sensitize these cells to the effects of drugs currently used in the treatment of haematological malignancies, such as bortezomib.

Mitochondrial FAO has been proposed as an alternative route for energy provision in tumor cells [8], [24]. Thus, it is plausible to hypothesize that pharmacological inhibition of FAO would result in increased glycolytic flux to sustain energy homeostasis in tumor cells. In support of this notion, Samudio et al. reported that etomoxir-mediated inhibition of FAO in leukemia cells increased the amount of lactate production and reduced pyruvate carboxylation as an adaptive mechanism to sustain ATP production [21]. However, we showed that etomoxir-mediated inhibition of FAO modestly increased the amount of lactate generated without altering glucose metabolism, suggesting that inhibition of FAO in myeloma cells does not result in an adaptive mechanism to sustain energy homeostasis. Finally, we measured glucose uptake and glucose utilization in cells that were not glucose-deprived. It is possible that etomoxir or orlistat might affect glucose transport and/or glucose utilization if those experiments had been performed in conditions of glucose deprivation. Further work is warranted to investigate the effects of orlistat and etomoxir on glucose metabolism under conditions of glucose deprivation.

Bearing in mind our results about the regulation of glucose and fatty acid metabolism in myeloma cells, it is reasonable to inquiry how two opposing pathways, such as fatty acid oxidation and de novo fatty acid synthesis, can function at the same time to drive cell growth in tumor cells. In view of the complexity of glucose and lipid metabolism in cancer cells, we hypothesize that acetyl-CoA derived from mitochondrial fatty acid oxidation may be used as a lipogenic substrate. In this way, fatty acid oxidation and de novo lipid biosynthesis can operate at the same time in myeloma. The rationale for this hypothesis is sustained by the work of Samudio et al. in leukemia cells [25]. The authors showed that under normoxic conditions exposure of leukemia cells to bone marrow-derived mesenchymal stromal cells promotes lactate accumulation in the culture medium and reduces mitochondrial membrane potential (i.e. the inability of uncoupled mitochondria to produce ATP). Because the consumption of glucose was not altered, the authors suggested that the accumulation of lactate resulted from reduced pyruvate metabolism. The authors proposed a shift to the oxidation of non-glucose carbon sources to maintain mitochondrial integrity and function. In this way, we proposed that fatty acid oxidation in myeloma cells might contribute as a source of acetyl-CoA, which can be used to replenish intermediaries in Krebs cycle and as a substrate for de novo lipid synthesis. Nonetheless, this hypothesis needs to be confirmed by experimental work.

The regulation of cell cycle progression and proliferation in myeloma cells is not fully understood. It has been shown that D-type cyclins are dysregulated in nearly all cases of multiple myeloma and monoclonal gammopathy of undetermined significance (MGUS) tumors [26]. Dysregulation of D-type cyclins have been proposed as an early and unifying oncogenic event in the pathogenesis of MM [26]. Thus, dysregulation of D-type cyclins expression by chromosomal alterations or unknown mechanisms may render cells more susceptible to proliferative stimuli resulting in expansion of the malignant clone. From the mechanistic point of view, upregulation of D-type cyclins would facilitate the binding and activation of CDK4 (or CDK6), which then phosphorylates and inactivates pRb so that E2F can induce cell cycle progression from G1 to S phase. In support of this notion, Quinn et al. have demonstrated that APRIL, a Proliferation Inducing Ligand that promotes survival and drug resistance in MM cell lines induces G1/S progression in primary MM cells. This effect was associated to the upregulation of cyclin D2, CDK4, CDK6 and phospho-pRb [27]. We showed that the anti-proliferative effect of etomoxir and orlistat was associated to the downregulation of proteins that regulate the pRb pathway, such as cyclin D2, CDK6 and phospho-pRb. Thus, our results reinforce the notion that inhibition of fatty acid metabolism represents a novel strategy to treating multiple myeloma.

Several approaches have been explored to target metabolic pathways in cancer cells, such as those related to limit the synthesis of macromolecules needed for cell growth or inhibition of metabolic pathways important to sustain bioenergetics [28]. Despite an increasing interest in targeting metabolic pathways for cancer therapy, very few molecules are currently in clinical trials [28]. Mounting evidences have shown that inhibitors of metabolic enzymes exhibited toxicity for cancer cells in vitro and in preclinical models of cancer. However, drug specificity, unwanted side effects and bioavailability are critical issues considered for the translation of preclinical data into clinical trials. Of note, the use of etomoxir in clinical trials [11], [12], [17], [18], and orlistat as an anti-obesity drug approved by the FDA [14], [29] have not been associated with serious adverse side effects. Interestingly, multiple myeloma is characterized by increased low-density lipoprotein (LDL) cholesterol clearance, which mediates an anti-apoptotic effect on myeloma cells [30], [31]. Thus, it is reasonable to propose the use of LDL-particles as a drug vehicle for etomoxir and orlistat delivery. This innovative approach would improve specificity and bioavailability of these drugs and might be used in combination with non-metabolic therapies, such as bortezomib, to enhance the toxicity of these compounds.

In conclusion, we demonstrated that inhibition of fatty acid metabolism is associated with an anti-proliferative and apoptotic effects in human myeloma cells. The mechanism of action by which etomoxir and orlistat reduced cell cycle progression and cell proliferation is mediated by inhibition of the pRb pathway. Finally, although further work is warranted to reveal the precise mechanisms by which orlistat and etomoxir is associated with anti-proliferative and apoptotic effects in myeloma cells, we propose that modulation of fatty acid metabolism by etomoxir and orlistat may represent a potential strategy for the treatment of human multiple myeloma.

## Materials and Methods

### Materials

Cell culture reagents (RPMI-1640 and fetal bovine serum) were from Invitrogen/Gibco, California, USA. [^3^H]-thymidine, [9,10-^3^H]-palmitic acid, 2-[^3^H]-deoxyglucose, D-[5-^3^H]-glucose, [^3^H]-H_2_O, [1,2-^14^C]-acetate and [D-^14^C]-glucose were from PerkinElmer, Massachusetts, USA. Etomoxir, orlistat, cerulenin and C75 were from Sigma, St. Louis, USA.

### Cell Culture

Human myeloma cell lines RPMI-8226, NCI-H929 and U-266B1 were purchased from the American Type Culture Collection (ATCC) and grown in RPMI-1640 medium supplemented with 10% (vol/vol) heat-inactivated fetal bovine serum (FBS), 100 U/mL penicillin G, and 100 µg/mL streptomycin. Cells were plate at density of 200,000 cells/mL. Culture media was replaced after 2 days of culture. The number of viable cells in suspension was determined using a haemocytometer by the trypan blue exclusion test.

### Fatty Acid Oxidation in Myeloma Cells

Fatty acid oxidation was measured as described previously [7]. Palmitate was preconjugated with essentially fatty acid–free bovine serum albumin (BSA) to generate stock solutions of 25% (wt/vol) BSA, 4 mM fatty acid in serum free medium. [^3^H]-Palmitate was added, and the stock solution was diluted into the final culture medium to give concentrations of 1.25% BSA, 0.1 mM (0.5 µCi/mL) fatty acid. Cells were incubated for 18 hours in the presence or absence of 50 µM etomoxir. The medium was then collected, and tritiated water was determined by the vapour-phase equilibration [7]. Cells were washed with ice-cold phosphate-buffered saline (PBS) and collected in 1 mL of 1N NaOH for measurement of protein content by the bicinchoninic acid method (Pierce, Illinois, USA).

### [^14^C]-Acetate Incorporation into Total Lipids in Myeloma Cells

De novo lipid synthesis was determined using [^14^C]-acetate according to Brown et al [7]. Cells were incubated in the presence or absence of 20 µM orlistat, plus [^14^C]-acetate (0.05 µCi/mL) for 24 hours. The cells were washed 3 times with 2 mL of ice-cold PBS and harvest in 90 µl of PBS; and after a total lipid extraction (according to Bligh and Dyer [32]), the radioactive content was determined. Protein content was measured as above.

### [^14^C]-Glucose Incorporation into Total Lipids in Myeloma Cells

De novo lipid synthesis was also determined using [^14^C]-glucose. Cells were incubated in the presence or absence of 20 µM cerulenin, 20 µM C75 or 20 µM orlistat, plus [^14^C]-glucose (0.5 µCi/mL) for 24 hours. The cells were washed 3 times with 2 mL of ice-cold PBS and harvest in 90 µl of PBS; and after a total lipid extraction (according to Bligh and Dyer), the radioactive content was determined. Protein content was measured as above.

### Glucose Metabolism Analysis in Myeloma Cells

Parameters of glucose metabolism in myeloma cells were measured after 18–24 hours incubation in the absence or presence of 50 µM etomoxir or 20 µM orlistat. Glucose utilization was determined as the production of tritiated water (as for fatty acid experiments) after incubation of myeloma cells for 10 minutes in the presence of [5-^3^H]-glucose (6.5 µCi/mL) [7]. Uptake of 2-[^3^H]-deoxyglucose (DOG; 10 µmol/L, 0.5 µCi/mL) was determined over 5 minutes after 18 or 24 hours of preincubation with etomoxir or orlistat respectively [33]. Protein content was measured as above.

### Measurement of Lactate Generation

Cells were incubated for 18–24 hours in the presence or absence of etomoxir or orlistat. The medium was then collected, and lactate content was determined using E-501 modular system from Roche Diagnostics analyzer with a linear range of standard lactate concentrations according to the procedures recommended by the manufacturer (Hoffmann-La Roche, Madrid, Spain).

### Cell Cycle Analysis

Cell cycle analysis was determined using propidium iodide nucleic acid staining method according to Cozar-Castellano et al [34]. Briefly, human myeloma cells were preincubated with 50 µM etomoxir or 20 µM orlistat for 18–24 hours as described above. Afterwards, cells were centrifuged, washed and resuspended in PBS containing 1% glucose. To fix the cells, we added 70% ethanol drop-wise while vortexing the cells at low speed. The fixed cells were then harvested and resuspended with phosphate-buffered saline containing 1% glucose (vol/vol), 50 µg/mL propidium iodide (Sigma), and 100 units/mL RNase A (Sigma). After incubation for 30 minutes at RT, cells were subjected to flow cytometry for cell cycle profile analysis.

### Apoptosis Quantification by FACS

Apoptosis was determined by flow cytometry (FACScalibur, Becton Dickinson equipped with an air-cooled argon ion laser emitting 15 mW at 488 nm.) as described previously [35]. Antibody used for staining was PE-conjugated rabbit anti-active caspase-3 (Becton Dickinson Biosciences, Madrid, Spain).

### Tritiated Thymidine Incorporation

Cell proliferation was quantified using the tritiated thymidine incorporation method according to Cozar-Castellano et al [36]. Myeloma cells were preincubated in the presence or absence of 50 µM etomoxir or 20 µM orlistat for 16–22 hours. Afterwards, cells were incubated in the presence of [^3^H]-thymidine (1 µCi/mL) for an additional 2 hours, and drug levels were maintained. To quantify the amount of [^3^H]-thymidine incorporation, cells were washed with ice-cold PBS, and the proteins and DNA precipitated in ice-cold 10% trichloroacetic acid (Sigma) on ice. The resulting precipitant was resuspended in 0.1 N NaOH for 30 minutes at 60°C, and neutralized with 0.1 N HCl. for liquid scintillation counting.

### Western Blotting

Cells were incubated for 18–24 hours in the presence or absence of 50 µM etomoxir, or 20 µM orlistat. Then, cell lysates were prepared using lysis buffer (125 mM Tris-HCl pH 7.5, 2% (vol/vol) SDS, 1 mM DTT, 1 mM PMSF and protease inhibitors (Protease Inhibitor cocktail, Sigma)). Proteins were resolved by 10% SDS-PAGE and electrotransferred onto PVDF membranes for immunoblotting by conventional means. After probing with phosphorylation-specific antibodies, the membranes were stripped and reprobed with antibodies against total kinase proteins and actin. Signals were detected by chemiluminescence (Immun-Star™ WesternC™ kit, Bio-Rad, Barcelona, Spain) using the Bio-Rad ChemiDoc XRS System. Four-to-six independent experiments were used for quantification of bands within the linear range with ImageJ software. Antibodies used were anti-p21, anti-p16, and anti-Cdk6 from Santa Cruz Biotechnology Inc., Heidelberg, Germany; anti-p27 and anti-pRb from Becton Dickinson, Biosciences; anti-Cdk4 and anti-phospho-pRb from Abcam, Cambridge, UK; anti-CyclinD1 and anti-CyclinD from Invitrogen; and Anti-actin from Sigma.

### Statistical Analysis

Data are expressed as means ± SEM. Statistical significance was determined by Student’s *t*-test or ANOVA. Differences were considered significant at p<0.05.
